# Early Ultrasound Identification of Cord Entanglement in Monochorionic Monoamniotic Twin Pregnancy

**DOI:** 10.3390/diagnostics11030520

**Published:** 2021-03-15

**Authors:** Anca Maria Panaitescu, Nicolae Gică, Radu Botezatu, Brîndușa Cimpoca, Alina Veduță, Gheorghe Peltecu, Anca Marina Ciobanu

**Affiliations:** 1 “Carol Davila” University of Medicine and Pharmacy, 050474 Bucharest, Romania; gica.nicolae@umfcd.ro (N.G.); radu.botezatu@umfcd.ro (R.B.); gheorghe.peltecu@gmail.com (G.P.); 2 Department of Obstetrics and Gynecology, Filantropia Clinical Hospital, 011171 Bucharest, Romania; brindusa.cimpoca@yahoo.com (B.C.); alina.veduta@gmail.com (A.V.); ciobanu.ancamarina@gmail.com (A.M.C.)

**Keywords:** monochorionic monoamniotic twin pregnancy, cord entanglement, fetal demise

## Abstract

Monochorionic monoamniotic pregnancy are considered high risk gestations and the fetal outcome is at times unpredictable. Correct diagnosis and counselling are extremely important, especially regarding the risk of unexpected fetal demise. We present the rare case of a monochorionic monoamniotic twin pregnancy with early identification of cord entanglement and the characteristic ultrasound findings in the first trimester of pregnancy.

Monochorionic monoamniotic twin pregnancy is rare, with an estimated prevalence of about 1 in 10,000 pregnancies, representing 1–5% of all monochorionic twins [1]. Correct diagnosis and counselling of parents are extremely important, especially regarding the risk of unexpected fetal demise. Without major abnormalities, the rates of fetal loss before 24 weeks are 20% [2] and the risk of perinatal mortality after 24 weeks ranges between 10–20% [2,3]. More recent data reported a lower risk of unexpected fetal death, of about 5–10%, after excluding cases of discordant structural anomalies, spontaneous miscarriage, or complications related to monochorionicity such as twin-to-twin transfusion syndrome (TTTS) [4]. After 32 weeks, the risk of sudden fetal death is 4% and usually involves both twins [5]. Cord entanglement has been evoked as the main cause of sudden death, although other mechanisms such as acute exsanguination through large umbilical anastomoses are likely to be involved, as cord entanglement is present in almost all monoamniotic twins and most of them have a good prognosis after 20 weeks despite this finding [3]. Diagnosis of chorionicity and amnionicity is easily established in the first trimester of pregnancy, along with other major structural defects [6]. Cord entanglement can be identified early in the first trimester using color Doppler and pulsed-wave Doppler by the simultaneous recording of two different heart rates [7] (Figure 1).

**Legend**

**Figure 1:**
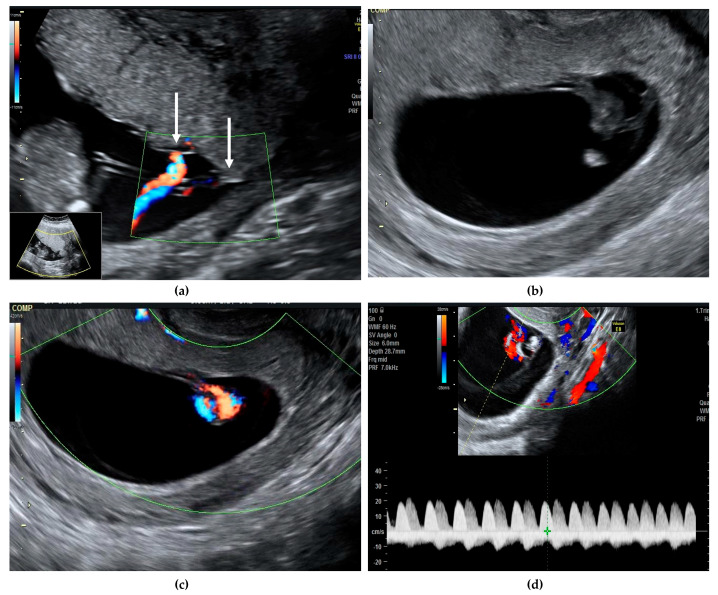
We present a case of a 30-year-old G2 P2 with a previous caesarean section, who presented in our unit at 11 weeks gestation for pregnancy confirmation after normal spontaneous conception. A viable monochorionic monoamniotic twin pregnancy was identified, and the common placenta and the cord insertions, very close to one another, were visualized (**a**, white arrows). The second ultrasound evaluation at 13 weeks did not reveal any structural abnormalities and the risk for aneuploidies was low (Supplementary Materials). Cord entanglement was suspected at this early gestational age, both in gray scale and color Doppler (**b**,**c**). Simultaneous registrations of two different heart rates also suggested the cord entanglement (**d**, represented by white and yellow arrows). The patient was counselled regarding the diagnosis, the possible complications, and the monitoring plan. The follow-up visit was planned for 16 weeks and both Doppler studies and growth were within normal ranges. From 16 weeks onwards, the follow-up scans were booked every second week. Unfortunately, the patient did not attend the 18 weeks scan, but she had a scan at 19 weeks. Sadly, double fetal demise was diagnosed. We acknowledge that this event was unpreventable and that routinely we recommend a scan every second week for reassurance. When fetal death occurs, this is an acute hemodynamic event, therefore previous scans are usually normal.

## Supplementary Materials

The following are available online at https://www.mdpi.com/2075-4418/11/3/520/s1, Monochorionic monomaniotic pregnancy early cord entanglemnet demonstration by ultrasound. S1—with color doppler; S2—in B mode.
